# Decolonizing implementation science: Centering community in the transition from intervention delivery to structural transformation

**DOI:** 10.1016/j.ghrp.2026.08.001

**Published:** 2026-08-05

**Authors:** Minmin Wang, Long Chen, Minghui Ren

**Affiliations:** a China Center for Health Development Studies, Peking University, China; b Department of Global Health, School of Public Health, Peking University, China; c Institute for Global Health, Peking University, China

**Keywords:** Decolonization, Implementation science, Global health equity, Community engagement

## Abstract

Colonial legacies in global health have entrenched structural inequities through unequal resource distribution, Western epistemic dominance, and the marginalization of Global South voices. Although implementation science aims to close the evidence–practice gap, its dominant frameworks often reinforce these hierarchies rather than dismantle them. Current approaches insufficiently consider communities’ positionality within global power structures, emphasize provider-level factors over social determinants, and overlook colonial histories and epistemic hierarchies. To advance decolonization, we propose a community-centred and equity-oriented recalibration of established implementation science frameworks. Specifically, Community, Reach, Effectiveness, Adoption, Implementation, Maintenance (CREAIM) is presented as an adaptation of the Reach, Effectiveness, Adoption, Implementation, and Maintenance RE-AIM framework, adding “Community” as a cross-cutting foundation that contextualizes Reach, Effectiveness, Adoption, Implementation, and Maintenance through explicit reflection on power, agency, and justice. In parallel, a decolonial recalibration of Consolidated Framework of Implementation Research (CFIR) emphasizes political economy analysis, locally led adaptation, equitable resource control, and co-created implementation processes. Together, these adaptations reposition implementation science from a technical instrument for intervention delivery into a vehicle for structural change. Embedding decolonial principles within implementation frameworks can reorient the field toward community leadership, epistemic justice, and sustainable global health equity.

## Background

Colonial legacies in global health have entrenched profound structural imbalances^1^, manifesting in multiple forms: deep inequalities in the distribution of resources and technologies^2^; the epistemic dominance of Western-centric knowledge systems that marginalize local health structures^3^; hierarchical power dynamics that deprive scholars in the Global South of leadership in research design^4^; and the systematic erosion of public health’s social mission under neoliberal market logics^5^.

Implementation science, which seeks to bridge the gap between evidence and practice and promote the uptake of evidence-based interventions, holds transformative potential to disrupt these colonial structures. Specifically, it may serve three critical functions: integrating local experience with scientific evidence to challenge epistemic hegemony; redistributing research leadership toward low- and middle-income countries (LMICs); and developing context-adapted interventions that shift from paternalistic aid models to equitable partnerships—ultimately advancing health justice through decolonization.

However, this potential remains constrained by the field’s own intellectual genealogy. Much of implementation science has drawn heavily on evidence-based medicine, behavioral science, organizational theory, and health services research. While these traditions have generated valuable theories, models, and frameworks, they may also embed assumptions of universality, linear knowledge translation, and externally defined evidence hierarchies. When applied uncritically in global health settings, such frameworks risk overlooking colonial histories, political economy, community agency, and locally grounded forms of knowledge.

We therefore approach from a situated standpoint, recognizing that implementation science is a field shaped by power, history, and unequal knowledge relations. In this context, existing framework tools may require not only technical refinement but also decolonial recalibration to better account for power, resource allocation, epistemic justice, and community leadership.

## Constrains in current implementation science framework

While implementation science has increasingly recognised the importance of context, equity, and adaptation, important limitations remain in how dominant frameworks are often operationalised in global health settings. Recent self-corrections within the field, including equity-oriented implementation frameworks, LMIC-focused adaptations of Consolidated Framework of Implementation Research (CFIR), indigenous implementation models, and participatory implementation approaches, have begun to address these gaps^6^, ^7^, ^8^. However, these advances remain unevenly integrated into mainstream framework use.

Prevailing models may reproduce linear and resource-centric assumptions when applied without critical adaptation. For example, RE-AIM has been widely used to evaluate Reach, Effectiveness, Adoption, Implementation, and Maintenance, and it has contributed substantially to implementation evaluation. Yet, if applied narrowly, “Reach” may be reduced to numerical coverage without asking who defines the target population or whether access is equitable; “Effectiveness” may prioritize biomedical or donor-defined outcomes over community-defined wellbeing; “Adoption” may focus on organizational or provider uptake rather than community ownership; “Implementation” may emphasize fidelity to externally designed protocols over locally led adaptation; and “Maintenance” may assess programme continuation without examining whether communities control resources or decision-making^9^. In this sense, the uncritical use of RE-AIM can risk reproducing top-down paradigms at the expense of community agency and contextual relevance.

Determinant frameworks such as Consolidated Framework for Implementation Research (CFIR contain constructs that can support decolonial practice, including attention to the outer setting, patient needs and resources, adaptability, available resources, engaging stakeholders, and implementation climate. LMIC-focused and equity-oriented adaptations of CFIR have further expanded attention to health systems, community engagement, and structural determinants^10^. Nevertheless, these practices remain insufficient for a decolonial agenda when power, political economy, epistemic authority, and resource control are treated as optional contextual variables rather than central implementation concerns.

Current framework tools exhibit structural blind spots: they tend to sidestep the political economy of resource allocation, the historical traumas of colonialism, and the enduring influence of epistemic dominance. Prevailing frameworks systematically marginalize local health practices that deviate from standardized Western paradigms. This knowledge-power disenfranchisement not only stifles innovation within endogenous knowledge systems but also induces attenuation in contextually grounded health interventions^7^.

## Reform of the frameworks to advance decolonization of global health

To advance the decolonization of global health, implementation science frameworks need to move beyond technical assessment of intervention delivery and more explicitly examine how power, knowledge, and resources are distributed throughout implementation.

The RE-AIM framework provides a valuable foundation for evaluating public health interventions, but when applied without an equity or decolonial lens, its dimensions may privilege externally defined priorities, provider-centred outcomes, and organisational uptake over community agency. We therefore propose Community, Reach, Effectiveness, Adoption, Implementation, Maintenance (CREAIM) as a recalibration of RE-AIM rather than a wholly new framework. In response, we propose an adapted framework—CREAIM to support this transformation (Fig. 1). In CREAIM, “Community” is not an additional evaluative domain but a cross-cutting foundation that informs all RE-AIM dimensions. Its role is to guide reflection across all dimensions, ensuring decolonial considerations remain central. For instance, *Reach* should identify excluded populations and structural drivers of marginalization, while *Effectiveness* must incorporate stratified outcome analyses to reveal disparities. *Adoption* should document implementation rates among eligible organizations, though contextual power dynamics affecting adoption decisions—such as local institutions’ real decision-making autonomy. Meanwhile, *Implementation* analysis must center on how leadership structures and resource flows perpetuate or challenge inequities, and *Maintenance* should track whether interventions sustain transformative decolonial practices. By centring the role of communities across all dimensions, CREAIM reorients implementation science toward a redistributive, justice-oriented approach—one that interrogates how power, knowledge, and resources are structured and restructured within local contexts.

**Fig. 1:**
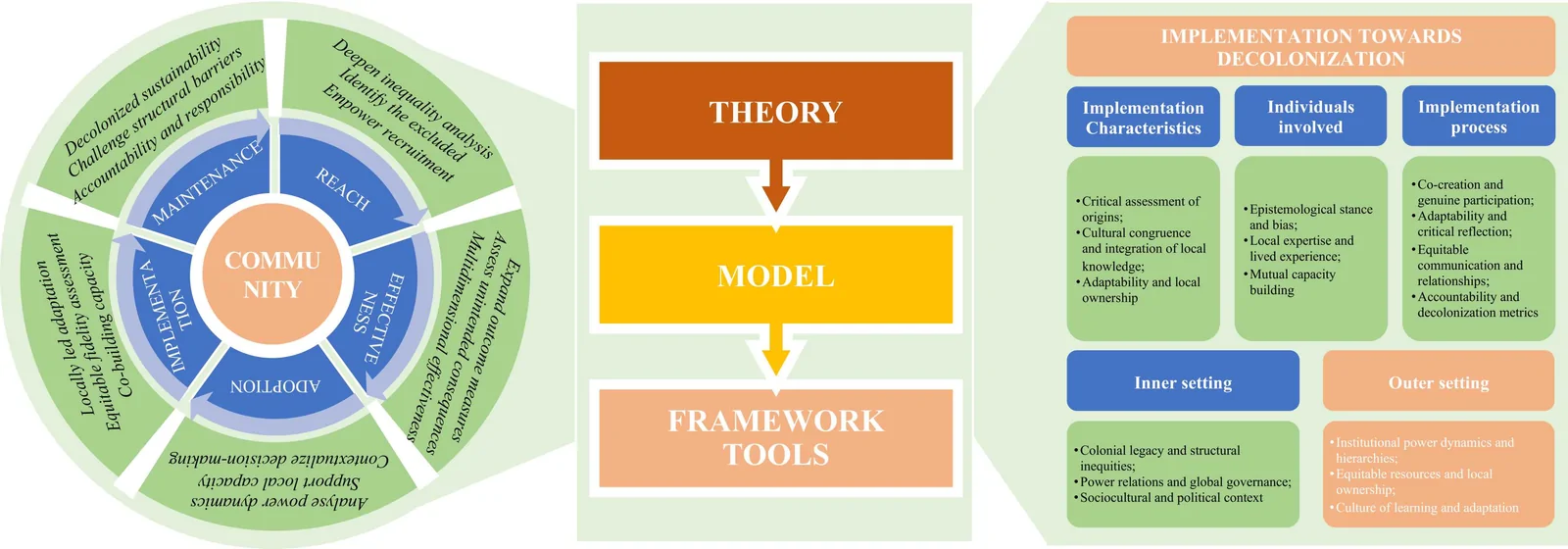
Reform the RE-AIM and CFIR frameworks to highlight the role of COMMUNITY to support global health decolonization.

The CFIR can also be recalibrated through a decolonial lens (Fig. 1). While domains such as *outer setting* nominally acknowledge structural determinants, these are often treated as immutable constraints, especially those linked to colonial histories and global inequities. A decolonial application of CFIR requires a deliberate reorientation:^1^ reconceptualizing the outer setting to explicitly engage with the political economy of health, and positioning them as modifiable structures that shape and can be reshaped by local agency;^2^ activating *intervention characteristics* to privilege locally led adaptation beyond superficial cultural tailoring;^3^ leveraging *inner setting* to prioritize equitable resource control over efficiency metrics;^4^ reframing *individual characteristics* to centre lived experience and co-produced capacity; and^5^ transforming the *process* domain into a co-creative praxis grounded in equitable engagement and decolonial metrics. Rather than expanding the framework with additional constructs, this reorientation subverts its constraint paradigm, and mobilizes CFIR’s existing architecture to disrupt embedded power asymmetries.

Several challenges should be noted. Community engagement can become tokenistic if communities are consulted but not given decision-making power. Funders and implementing agencies may limit local adaptation through rigid protocols, timelines, or predefined indicators. Measuring power redistribution, epistemic justice, and community agency is methodologically difficult and may require mixed methods, participatory evaluation, and locally developed indicators. Tensions may also arise between fidelity to evidence-based interventions and adaptation to local priorities.

## Conclusions

Decolonization must be embedded across the entire continuum of global health interventions, from upstream processes of knowledge generation and design to localized implementation, culture-sensitive evaluation, and community-engaged dissemination. Yet current implementation science overwhelmingly prioritizes the delivery phase, often neglecting how upstream (research framing, funding flows) and downstream (knowledge translation, policy uptake) processes entrench structural inequities. A decolonial approach requires sustained andparticipatory engagement throughout the full intervention cycle. Such reforms reposition implementation science from a technical instrument focused on intervention delivery to a transformative vehicle capable of addressing structural inequities, and aligning it more closely with the core agenda of global health decolonisation. In this comment, we propose CREAIM as a decolonial recalibration of the established RE-AIM framework, and the integration of decolonial content into existing models like CFIR. Meaningful change demands a more fundamental re-examination of the theoretical foundations and conceptual models that underpin the field. Only through sustained critical engagement with its theories and models can implementation science contribute to the global health decolonization agenda.

## Ethics approval and consent to participate

Not applicable.

## Funding

The work is supported by the Peking University Health Science Center Discipline Development Project (BMU2026RCZX108).

## CRediT authorship contribution statement

**Minmin Wang:** Writing – original draft, Data curation, Conceptualization. **Long Chen:** Writing – original draft, Conceptualization. **Minghui Ren:** Writing – review & editing, Supervision, Conceptualization.

## Declaration of Generative AI and AI-assisted technologies in the writing process

During the preparation of this work, the authors used ChatGPT−5.5 in order to improve language and readability. After using this service, the authors reviewed and edited the content as needed and take(s) full responsibility for the content of the publication.

## Declaration of Competing Interest

The authors declare that they have no known competing financial interests or personal relationships that could have appeared to influence the work reported in this paper.

## Data Availability

No data was used for the research described in the article.
